# A Stepping Trail Making Test as an Indicator of Cognitive Impairment in Older Adults

**DOI:** 10.3390/jcm9092835

**Published:** 2020-09-02

**Authors:** Yosuke Osuka, Hunkyung Kim, Yutaka Watanabe, Yu Taniguchi, Narumi Kojima, Satoshi Seino, Hisashi Kawai, Ryota Sakurai, Hiroki Inagaki, Shuichi Awata, Shoji Shinkai

**Affiliations:** 1 Research Team for Promoting Independence and Mental Health, Tokyo Metropolitan Institute of Gerontology, Tokyo 173-0015, Japan; kimhk@tmig.or.jp (H.K.); nkojima@tmig.or.jp (N.K.); inagaki@tmig.or.jp (H.I.); awata@tmig.or.jp (S.A.); 2 Faculty of Dental Medicine, Hokkaido University, Hokkaido 060-8586, Japan; ywata@den.hokudai.ac.jp; 3 Japan Environment and Children’s Study Programme Office, National Institute for Environmental Studies, Ibaraki 305-0053, Japan; taniguchi.yu@nies.go.jp; 4 Research Team for Social Participation and Community Health, Tokyo Metropolitan Institute of Gerontology, Tokyo 173-0015, Japan; seino@tmig.or.jp (S.S.); sakurair@tmig.or.jp (R.S.); 5 Research Team for Human Care, Tokyo Metropolitan Institute of Gerontology, Tokyo 173-0015, Japan; hkawai@tmig.or.jp; 6 Graduate School of Nutrition and Health Science, Kagawa Nutrition University, Saitama 350-0288, Japan; shinkai.shoji@eiyo.ac.jp

**Keywords:** integrated discrimination improvement, motor-cognitive dual-task test, net reclassification improvement

## Abstract

This study aimed to examine the concurrent validity of a novel motor-cognitive dual-task test, the Stepping Trail Making Test (S-TMT), as an indicator of cognitive impairment (CI), and compare its screening performance to that of motor or cognitive tests alone. This was a population-based cross-sectional study including 965 Japanese adults aged ≥ 70 years. To measure the time taken to perform the S-TMT, the participants were instructed to step on 16 numbers in sequence as quickly and accurately as possible. Motor and cognitive functions were assessed by gait speed and TMT part A (TMT-A), respectively. Participants were classified into CI (< 24 points), mild CI (MCI, 24–27 points), and intact cognition (> 27 points) categories based on their Mini-Mental State Examination score. Binary logistic regression models showed that the addition of the S-TMT to the covariates model gave the highest discrimination index (c-statistics), and significantly improved reclassification indices (net reclassification improvement and integrated discrimination improvement) for screening both CI and MCI compared to those of gait speed or TMT-A alone. These results show that S-TMT has a concurrent validity as a dual-task test for screening CI and MCI and better discrimination and reclassification performance than motor or cognitive tests alone in older adults.

## 1. Introduction

In 2020, the Lancet commission reported 12 modifiable risk factors for dementia [1]. These factors include less education in early-life, hearing loss, traumatic brain injury, hypertension, excessive alcohol consumption, and obesity in mid-life, and smoking, depression, social isolation, physical inactivity, air pollution, and diabetes in later-life. Modifying these factors could either prevent or delay up to 40% of dementias [1]. A higher level of physical activity and dietary variety (e.g., the Mediterranean diet) in mid- and later-life has been reported to prevent dementia and cognitive decline through decreasing the risk of obesity, diabetes, and cardiovascular disease [2]. The underlying mechanisms of dementia prevention based on a favorable lifestyle of physical activity and varied diet may be explained by the interaction between motor and cognitive functions. Although the causal ordering of the two functions remains under debate, motor function parameters, such as handgrip strength [3,4,5], chair stand [5,6], balance [4,6], and gait speed [7,8,9,10,11], have been proposed as indicators of cognitive decline in older adults. Notably, gait speed is more strongly associated with fluid cognition than other motor function parameters [12], and is a good predictor of dementia [13,14]. Thus, adding a gait speed test to the standard screening is very useful for early detection of suspected dementia among community-dwelling older adults, especially in primary care settings or developing countries [13].

Gait speed has good feasibility for screening cognitive impairment (CI) and mild CI (MCI) in clinical settings. It can be assessed more easily than psychophysiological or brain morphological measurements since it is inexpensive and does not require specialized techniques [15]. However, preferred gait speed (i.e., walking at a comfortable pace) is an automatic motor task for physically and cognitively robust older adults, indicating that it hardly stimulates cognitive function in that population [16]. Consequently, some studies have argued that although the association between gait speed and cognition function was significant, the association was weak [17] and gait speed alone may lack sensitivity and specificity for screening CI [18]. The Canadian Consortium on Neurodegeneration in Aging has indicated the importance of measuring both motor and cognitive functions for predicting CI, and proposed a complementary battery of assessments, including gait speed, Trail Making Test (TMT), and dual-task gait [19].

Sheridan et al. reported that although executive and neuropsychological functions were significantly associated with gait performance during a cognitively challenging walking task, they were not associated with simple gait speed [16]. These results suggest that the relationship between cognitive function and gait performance may be strengthened during a cognitively challenging task. Therefore, a motor task during a cognitively challenging task may be a better indicator of CI and MCI than a motor or cognitive task alone. Alexander et al. were the first to propose a Walking Trail Making Test (WTMT), which combined a visual tracking task assessed by a standard neuropsychological test (i.e., the TMT part A: TMT-A) with a walking task as a clinical test of stepping accuracy under conditions of increased cognitive and visual demand [20]. Subsequently, Yamada et al. modified this to create the Trail Walking Test (TWT), and identified that it was useful for predicting the risk of falling in older adults [21]. Klotzbier et al. and Perrochon et al. further focused on the usefulness of the WTMT and TWT as screening tests for the early detection of MCI [22,23]. To expand on these previous studies, we recently developed the Stepping TMT (S-TMT) and examined its reliability and validity among 1224 community-dwelling older Japanese women without physical disability, neurodegenerative diseases, or visual impairments [24]. The study identified that the S-TMT was a reliable dual-task test comprising stepping for motor function and visual-dependent execution for cognitive function [24]. The S-TMT has a simplified measurement process and requires less space compared to both the WTMT and TMT, suggesting that it could be a practical and useful test for screening CI and MCI. However, a concurrent validity of the S-TMT as an indicator of CI and MCI and its screening performance has yet to be identified.

This study was undertaken to examine a concurrent validity of the S-TMT as an indicator of CI and MCI and compare its screening performance with motor or cognitive tests alone among older Japanese adults. We hypothesized that a longer S-TMT time would be significantly associated with a higher probability of CI and MCI, and that the S-TMT would have a better screening performance for detecting CI and MCI than motor and cognitive tests alone.

## 2. Experimental Section

### 2.1. Study Design and Setting

This study was conducted as part of a population-based cross-sectional study (the Takashimadaira Study). A complete survey was conducted at the regional public facility in Takashimadaira, Itabashi ward in Tokyo, Japan, between October and December of 2016. The primary purpose of the Takashimadaira Study was to observe the prevalence of dementia and identify psychological and physical factors related to dementia among metropolitan-dwelling older adults. The survey was administered in two steps: a mailed interview survey and an on-site survey.

### 2.2. Participants

The initial interview survey was mailed to all older adults aged ≥ 70 years with an address in Takashimadaira, as listed in the basic resident register (*n* = 7614). A total of 5430 individuals responded to the mailed interview survey (response rate: 71.3%). Thereafter, a letter was sent to all respondents inviting them to participate in an on-site survey. Ultimately, 1248 individuals participated in both steps of the study (participation rate: 16.4%). We excluded participants who had (1) a physical disability, depression, Parkinson’s disease, or visual impairment (*n* = 124, 9.9%), and (2) missing data in any of the variables (*n* = 159, 12.7%). Accordingly, we included 965 participants in the final analysis (data inclusion rate: 77.3%). This study was conducted in accordance with the Declaration of Helsinki and the study protocol was approved by our institutional Ethics Committee. All participants provided written informed consent.

### 2.3. Measurements

#### 2.3.1. CI and MCI

CI and MIC were assessed by the Japanese version of the Mini-Mental State Examination (MMSE-J), which was developed on the basis of a contract with the original MMSE publisher [25]. The MMSE has validity and reliability for assessing the cognitive state [26], and is widely used to screen for CI and MCI [25,27]. Via face-to-face interview, trained nurses or psychologists asked the participants to perform or answer 11 cognitive tasks or questions respectively, which corresponded to five cognitive domains (orientation, memory, language, attention, and visuospatial abilities). MMSE-J scores ranged from 0 to 30 points, with higher scores indicating better cognitive function. Participants were classified into CI (<24 points), MCI (24–27 points), and intact cognition (>27 points) categories based on the MMSE score [25,27].

#### 2.3.2. Motor-Cognitive Dual-Task

A motor-cognitive dual-task was assessed using the S-TMT. A previous study of its reliability and construct validity study has described the method of the S-TMT measurement in detail [24]. Briefly, participants were instructed to step on a sequence of numbered (1–16) squares (25 × 25 cm) positioned on a square rubber mat (1 m^2^) as quickly and accurately as possible, and the time taken from start call to stepping on the last number (16) was measured. The measurement time included the time for correcting errors. The measurement was performed in a single trial. The S-TMT has been shown to have a good intra-rater reliability (intra-class correlation coefficient and 95% confidence interval: 0.82, 0.68–0.90) [24].

#### 2.3.3. Single Motor Task

The single motor task was assessed by the gait speed test. Gait speed was measured as the time taken to walk 5 m (between markers at 3 and 8 m of an 11 m walking path) measured with a stopwatch [28]. Participants were instructed to walk at their usual (i.e., preferred) speed. Gait speed measurements were performed in a single trial.

#### 2.3.4. Single Cognitive Task

The single cognitive task was assessed using the TMT-A, which assesses attention, processing speed, and in particular, visuoperception [29,30]. Participants were asked to draw lines, as quickly as possible, sequentially connecting consecutively numbered (1–25) circles randomly arranged on a paper. TMT-A was performed in a single trial after one practice on numbers from one to eight. The total TMT-A time included the time for correcting errors.

#### 2.3.5. Covariates

Age, sex, years of education, number of housemates, medical history, drinking and smoking statuses, body mass index (BMI), depression status, and handgrip strength were investigated as potentially relevant variables. Participants who did not live with a housemate were defined as living alone. All participants were asked whether they had a medical history of hypertension, stroke, heart disease, diabetes mellitus, hyperlipidemia, or osteoporosis in the past year. Current smoking and drinking statuses were classified into two categories: “current” as “yes” and “past or almost never” as “no.” BMI was calculated by dividing weight (kg) by the square of height (m). Depression status was assessed by the short-version of the Geriatric Depression Scale (GDS) [31]. We assessed handgrip strength using a Smedley-type dynamometer. Trained testers instructed the participants to stand naturally and grip the device with their dominant hand as strongly as possible [28]. Handgrip strength was measured twice, and the highest measurement value was used in analyses.

### 2.4. Statistical Analysis

First, Spearman’s rank correlation coefficient (*ρ*) was calculated to examine univariate associations between MMSE score and S-TMT time, gait speed, and TMT-A time. Second, we adjusted for potential confounders (age, sex, years of education, BMI, stroke, heart disease, diabetes mellitus, GDS score, handgrip strength) in multiple regression models that included the MMSE score as a dependent variable and the S-TMT time, gait speed, or TMT-A time as independent variables. Third, to examine if the S-TMT time, gait speed, and TMT-A time were associated with the probability of CI in participants with MMSE scores < 27 points, and MCI in participants with MMSE scores > 24 points, we applied binary logistic regression models that included either CI or MCI as a dependent variable, and the S-TMT time, gait speed, or TMT-A time as independent variables. After adjustment for confounders, the odds ratios and 95% confidence intervals for CI and MCI were calculated for the three independent variables. Next, receiver operating characteristic (ROC) analysis was applied to show the discrimination performance of the S-TMT time, gait speed, and TMT-A for screening CI and MCI. The area under the curve (AUC) and c-statistics were calculated by plotting sensitivity and 1–specificity [32]. The cut-off values of the S-TMT time, gait speed, and TMT-A time for screening CI and MCI were determined using the Youden index. Finally, continuous net reclassification improvements (cNRIs) and integrated discrimination improvements (IDIs) were calculated to examine whether the addition of S-TMT time to the covariates model showed better reclassification performance for screening CI and MCI than that of gait speed or TMT-A time [33,34]. The cNRI can be interpreted as the percentage of participants with correct movement in estimated prediction probabilities. The IDI is a continuous measure that evaluates the improvement in average sensitivity minus the change in average 1–specificity. The cNRIs and IDIs indices were computed by the PredictABEL command in R software. All analyses were performed with IBM SPSS version 25.0 (IBM Corp., Armonk, NY, USA) and R version 3.6.2 (The R Foundation for Statistical Computing Platform, Vienna, Austria). *p* values < 0.05 were considered statistically significant.

## 3. Results

### 3.1. Characteristics of Study Participants

Table 1 shows the characteristics of the included participants. The median and interquartile range of the main variables were as follows: S-TMT time, 16.1 (12.9–20.3) s, gait speed, 1.3 (1.1–1.4) m/s, TMT-A time, 42.0 (34.0–54.0) s, and MMSE score, 28.0 (26.0–29.0) points. The number and percent of participants with CI, MCI, and intact cognition were 61 (6.3%), 345 (35.9%), and 559 (57.9%), respectively.

### 3.2. Associations between MMSE Score and the S-TMT Time, Gait Speed, or TMT-A Time

The S-TMT time, gait speed, and TMT-A time were significantly associated with MMSE score (S-TMT, *ρ* = −0.28, *p* < 0.01; gait speed, *ρ* = 0.14, *p* < 0.01; TMT-A, *ρ* = −0.23, *p* < 0.01). Even after adjusting for potential confounders, multiple linear regression analyses showed that the longer S-TMT time, slower gait speed, and longer TMT-A time were significantly associated with lower MMSE score (S-TMT, *β* = −0.29, *p* < 0.01; gait speed, *β* = 0.07, *p* = 0.04; TMT-A, *β =* −0.20, *p* < 0.01).

### 3.3. Discrimination Performance of the S-TMT, Gait Speed, and TMT-A for Screening CI and MCI

Table 2 shows the AUCs and cut-off values of the S-TMT time, gait speed, and TMT-A time for screening CI and MCI. The AUCs of the S-TMT for discriminating CI and MCI were higher than those of gait speed and TMT-A time. Table 3 shows the adjusted odds ratios and c-statistics of the S-TMT time, gait speed, and TMT-A time for screening CI and MCI. A longer S-TMT time, slower gait speed, and longer TMT-A time had significantly higher adjusted odds ratios for screening CI; however, the c-statistics of the covariates plus S-TMT model were higher than those of the covariates plus either gait speed or TMT-A time models. For screening MCI, only a longer-S-TMT time had a significantly higher adjusted odds ratio, and the c-statistic of the covariates plus S-TMT model was higher than those of the covariates plus either gait speed or TMT-A time models.

### 3.4. Comparison of the Reclassification Performance of Gait Speed, TMT-A, and S-TMT for Screening CI and MCI

Table 4 summarizes the comparisons of reclassification indices for screening CI and MCI between the addition of S-TMT time or either gait speed or TMT-A time to the covariates model. All cNRI and IDI indices for screening CI and MCI were significantly higher in the model of covariates with the S-TMT than in the model of covariates with the gait speed or TMT-A, indicating that the S-TMT improved reclassification for screening CI and MCI, compared to the gait speed or TMT-A.

## 4. Discussion

This study showed that the S-TMT time, gait speed, and TMT-A time were significantly associated with the MMSE score; however, the S-TMT time was more strongly associated with the MMSE score than either gait speed or TMT-A time. Additionally, the S-TMT exhibited a better discrimination and reclassification performance for screening both CI and MCI compared to the gait speed or TMT-A. These results support our hypothesis that S-TMT has concurrent validity as an indicator of CI and MCI and a better screening performance for detecting CI and MCI than gait speed and TMT-A time alone in older adults.

This study was the first to identify that a longer S-TMT time was significantly associated with a lower MMSE score in older adults. This association was robust, as the association remained unchanged even after adjusting for confounding variables. Our previous study of the construct validity reported that the S-TMT was significantly associated with the Symbol Digit Substitution Task, TMT-A and -B as cognitive domain, and gait speed as motor domain, indicating that the S-TMT could assess two components: cognitive function requiring visuoperceptual ability, visual attention, and visuospatial memory, and a motor function that requires agility and stable balance for stepping [24]. Thus, good cooperation between high vision-dependent execution function to locate upcoming numbers and high mobility function to step with stability would be required in order to decrease the S-TMT time. The interdependence between motor and cognitive function in older people is demonstrated as the fact that lower motor function is more prevalent in people with CI and MCI [35,36]. Thus, assessment of motor-cognitive interaction using the S-TMT could represent a new methodological approach for screening CI and MCI in community-dwelling older adults. In clinical practice, patients whose S-TMT time is ≥ 18.5 s and ≥ 15.7 s could be considered to have possible CI and MCI, respectively. However, physicians should note that the S-TMT alone had a low discrimination performance for MCI (AUC = 0.60); therefore, we recommend using it in combination with a general screening tool for MCI.

It remains unclear if motor tests during a cognitively challenging task are best indicators of CI and MCI among community-dwelling older adults [9,18]. Here, we found that a motor task (stepping task) during a cognitively challenging task (visual tracking task) was a better indicator of CI and MCI than motor or cognitive tasks alone. Preferred gait speed is recognized as an almost involuntary activity, but it requires the coordination of several neurological systems, including motor, sensory, and cerebellar activities [14]. Gait performance depends on executive function, as it uses common brain networks and shares resources with cognitive performance in order to control future steps [37]. This may support slow gait speed as a predictor of cognitive decline and dementia. However, the nature of gait as an automated performance task has led some studies to speculate that the degree of association between gait and cognitive function is insufficient for estimating the cognitive capacity [17,18]. Montero-Odasso et al. recently demonstrated that single-task gait speed was not associated with progression to dementia; however, dual-task gait cost—the magnitude of change in gait during dual-task performance due to a concurrent cognitive challenge—was associated with an increased risk of progression to dementia among older adults with MCI. Thus, gait speed alone may not have sufficient sensitivity for a higher risk of progression to dementia. Conversely, dual-task gait was proposed as a potentially useful clinical motor marker for predicting future dementia [38]. Furthermore, Klotzbier et al. attempted to discriminate the MCI using the TWT and reported that TWT with higher cognitive load, such as walking on targets with increasing sequential number and letters (i.e., 1-A-2-B-3-C…), would be a potential marker for early MCI detection [22]. Our results support these arguments, because the S-TMT time was more strongly associated with MMSE score and had better discrimination and reclassification indices for screening CI and MCI than gait speed or TMT-A alone. Although the specific mechanism of S-TMT superiority is uncertain, when a motor (stepping) task is concurrently performed with a cognitive (trailed number) task, motor control and cognition are shared. This common brain network (e.g., prefrontal and temporal areas) causes overload and confusion. This is especially remarkable in individuals with CI who exhibit reduced cognitive reserve [39]. The current study has expanded on this to show that the motor-cognitive dual-task test may also be more useful for the discrimination of MCI than motor or cognitive tasks alone, since only the S-TMT time had a significantly higher adjusted odds ratio (OR) and improved reclassification performance for screening MCI.

The S-TMT may be useful for further emphasizing and assessing cognitive aspects compared to single motor function tests. Additionally, it can be performed more conveniently than general neuropsychological tests, making it possible to reduce missing values and shorten the measurement time. Indeed, our data showed that the S-TMT had fewer missing values (missing value rate, 0.8% vs. 5.9%) and shorter measurement times (see Table 1) than the TMT-A. Although the current study did not compare the usefulness of the S-TMT for screening CI and MCI with that of other motor-cognitive dual-task tests (e.g., WTMT, TWT, and dual-task gait), the S-TMT has been improved to simplify the measurement process and use less measurement space (1 m^2^). These advantages highlight that the S-TMT could be a useful and practical screening tool in the clinical setting, especially in community primary care and developing countries.

This study also had some limitations. First, although the study results supported our hypothesis, it remains unclear whether S-TMT time can predict cognitive decline, given the cross-sectional design of the study. Second, a majority of the target population did not participate in our study (6366/7614, 83.6%). Therefore, the included population may exhibit a healthy volunteer effect—participants might have been healthier than the general population, limiting the generalizability of our results to an overall older adult population. Third, unmeasured potential confounders might be associated with both S-TMT and CI or MCI. Therefore, additional studies are necessary to adjust other confounding factors potentially influencing the association between S-TMT time and global cognition. Fourth, the detailed mechanism of better CI and MCI detection by motor and cognitive dual-task test compared to motor and cognitive tests alone remains unclear. Finally, the current study operationally defined CI and MCI using the MMSE score, but their accurate diagnosis requires the completion of more adequate neuropsychological tests. Future studies should examine the diagnostic performance of the S-TMT for detecting more reliably validated CI and MCI.

## 5. Conclusions

A longer S-TMT time was significantly associated with a higher probability of CI and MCI, indicating that S-TMT time has a concurrent validity as an indicator of CI and MCI. Additionally, the S-TMT performed better at screening for CI and MCI than motor and cognitive function tests alone among older Japanese adults. These results suggest that the S-TMT could be a useful and practical test for screening CI and MCI in the clinical setting. A prospective longitudinal study is required to confirm whether this indicator can also predict cognitive decline.

## Figures and Tables

**Table 1:** Characteristics of study participants.

Age, years	77 (74–81)
Sex, men/women	402/563
Body mass index, kg/m^2^	22.9 (21.1–25.0)
Years of education	12 (12–15)
Living alone, yes	383 (39.7%)
Hypertension, yes	507 (52.5%)
Stroke, yes	79 (8.2%)
Heart disease, yes	194 (20.1%)
Diabetes mellitus, yes	139 (14.4%)
Hyperlipidemia, yes	383 (39.7%)
Osteoporosis, yes	240 (24.9%)
Handgrip strength, kg	26.0 (21.0–31.0)
GDS score, point	3.0 (1.0–5.0)
Smoker, yes	61 (6.3%)
Drinker, yes	422 (43.7%)

Note: Data are shown as median (interquartile range) or *n* (%). GDS, Geriatric and Depression Scale.

**Table 2:** Discrimination performance and cut-off values of the S-TMT, gait speed, and TMT-A for cognitive impairment and mild cognitive impairment screening.

	AUC (95% CI)	Cut-off Values	Sensitivity (%)	Specificity (%)
Discrimination of cognitive impairment in participants with MMSE scores < 27 points (*n* = 406)				
S-TMT, s	0.75 (0.68–0.82)	18.4/18.5	80.3	63.2
Gait speed, m/s	0.61 (0.54–0.69)	1.07/1.08	41.0	76.5
TMT-A, s	0.72 (0.65–0.79)	46.0/47.0	82.0	60.0
Discrimination of mild cognitive impairment in participants with MMSE scores > 24 points (*n* = 904)				
S-TMT, s	0.60 (0.56–0.64)	15.6/15.7	61.4	55.1
Gait speed, m/s	0.55 (0.51–0.58)	1.17/1.18	36.8	73.3
TMT-A, s	0.57 (0.54–0.61)	44.0/45.0	46.7	63.7

AUC, area under the curve; CI, confidence interval; S-TMT, Stepping Trail Making Test; TMT-A, Trail Making Test A; MMSE, Mini-Mental State Examination.

**Table 3:** Adjusted odd ratios (ORs) and discrimination performance for cognitive impairment and mild cognitive impairment screening between covariates models with the addition of either S-TMT, gait speed, or TMT-A.

	Adjusted ORs (95% CI)	C-statistics (95% CI)
Discrimination of cognitive impairment in participants with MMSE scores < 27 points (*n* = 406)		
Covariates model		0.66 (0.58–0.73)
+ S-TMT, s	1.12 (1.07–1.17)	0.77 (0.71–0.83)
+ Gait speed, m/s	0.25 (0.07–0.95)	0.67 (0.59–0.74)
+ TMT-A, s	1.02 (1.01–1.04)	0.73 (0.66–0.80)
Discrimination of mild cognitive impairment in participants with MMSE scores > 24 points (*n* = 904)		
Covariates model		0.64 (0.60–0.67)
+ S-TMT, s	1.03 (1.01–1.06)	0.65 (0.61–0.69)
+ Gait speed, m/s	1.02 (0.52–1.99)	0.64 (0.60–0.67)
+ TMT-A, s	1.00 (1.00–1.01)	0.64 (0.60–0.68)

Covariates model included age, sex, years of education, body mass index, stroke, heart disease, diabetes mellitus, geriatric depression scale score, and handgrip strength. S-TMT, Stepping Trail Making Test; TMT-A, Trail Making Test A; ORs, odds ratios; CI, confidence interval; MMSE, Mini-Mental State Examination.

**Table 4:** Comparisons of reclassification indices for screening cognitive impairment and mild cognitive impairment between the addition of S-TMT or either gait speed or TMT-A to the covariates model.

	cNRI (95% CI)	*p*	IDI (95% CI)	***p***
Reclassification of the cognitive impairment in the participants with MMSE scores < 27 points (*n* = 406)				
Covariates + Gait speed model	Reference		Reference	
vs. covariates + S-TMT model	0.54 (0.28–0.81)	<0.01	0.08 (0.04–0.12)	<0.01
Covariates + TMT-A model	Reference		Reference	
vs. covariates + S-TMT model	0.30 (0.03–0.57)	0.03	0.05 (0.01–0.09)	0.02
Reclassification of the mild cognitive impairment in the participants with MMSE scores > 24 points (*n* = 904)				
Covariates + Gait speed model	Reference		Reference	
vs. covariates + S-TMT model	0.18 (0.05–0.31)	<0.01	0.01 (0.00–0.01)	0.02
Covariates + TMT-A model	Reference		Reference	
vs. covariates + S-TMT model	0.21 (0.08–0.34)	<0.01	0.01 (0.00–0.01)	0.04

Covariates model included age, sex, years of education, body mass index, stroke, heart disease, diabetes, geriatric depression scale score, and handgrip strength. CI, confidence interval; cNRI, continuous net reclassification improvement; IDI, integrated discrimination improvement; S-TMT, Stepping Trail Making Test; TMT-A, Trail Making Test A; MMSE, Mini-Mental State Examination.

## References

[1] Livingston G., Huntley J., Sommerlad A., Ames D., Ballard C., Banerjee S., Brayne C., Burns A., Cohen-Mansfield J., Cooper C. (2020). Dementia prevention, intervention, and care: 2020 report of the Lancet Commission. Lancet.

[2] (2019). Risk Reduction of Cognitive Decline and Dementia: WHO Guidelines.

[3] Alfaro-Acha A., Al Snih S., Raji M.A., Kuo Y.F., Markides K.S., Ottenbacher K.J. (2006). Handgrip strength and cognitive decline in older Mexican Americans. J. Gerontol. A Biol. Sci. Med. Sci..

[4] Malmstrom T.K., Wolinsky F.D., Andresen E.M., Miller J.P., Miller D.K. (2005). Cognitive ability and physical performance in middle-aged African Americans. J. Am. Geriatr. Soc..

[5] Auyeung T.W., Lee J.S., Kwok T., Woo J. (2011). Physical frailty predicts future cognitive decline—A four-year prospective study in 2737 cognitively normal older adults. J. Nutr. Health Aging.

[6] Rosano C., Simonsick E.M., Harris T.B., Kritchevsky S.B., Brach J., Visser M., Yaffe K., Newman A.B. (2005). Association between physical and cognitive function in healthy elderly: The health, aging and body composition study. Neuroepidemiology.

[7] Marquis S., Moore M.M., Howieson D.B., Sexton G., Payami H., Kaye J.A., Camicioli R. (2002). Independent predictors of cognitive decline in healthy elderly persons. Arch. Neurol..

[8] Fitzpatrick A.L., Buchanan C.K., Nahin R.L., Dekosky S.T., Atkinson H.H., Carlson M.C., Williamson J.D. (2007). Ginkgo Evaluation of Memory Study, I. Associations of gait speed and other measures of physical function with cognition in a healthy cohort of elderly persons. J. Gerontol. A Biol. Sci. Med. Sci..

[9] Deshpande N., Metter E.J., Bandinelli S., Guralnik J., Ferrucci L. (2009). Gait speed under varied challenges and cognitive decline in older persons: A prospective study. Age Ageing.

[10] Taniguchi Y., Yoshida H., Fujiwara Y., Motohashi Y., Shinkai S. (2012). A prospective study of gait performance and subsequent cognitive decline in a general population of older Japanese. J. Gerontol. A Biol. Sci. Med. Sci..

[11] Mielke M.M., Roberts R.O., Savica R., Cha R., Drubach D.I., Christianson T., Pankratz V.S., Geda Y.E., Machulda M.M., Ivnik R.J. (2013). Assessing the temporal relationship between cognition and gait: Slow gait predicts cognitive decline in the Mayo Clinic Study of Aging. J. Gerontol. A Biol. Sci. Med. Sci..

[12] Clouston S.A., Brewster P., Kuh D., Richards M., Cooper R., Hardy R., Rubin M.S., Hofer S.M. (2013). The dynamic relationship between physical function and cognition in longitudinal aging cohorts. Epidemiol. Rev..

[13] Beauchet O., Annweiler C., Callisaya M.L., De Cock A.M., Helbostad J.L., Kressig R.W., Srikanth V., Steinmetz J.P., Blumen H.M., Verghese J. (2016). Poor Gait Performance and Prediction of Dementia: Results From a Meta-Analysis. J. Am. Med. Dir. Assoc..

[14] Quan M., Xun P., Chen C., Wen J., Wang Y., Wang R., Chen P., He K. (2017). Walking Pace and the Risk of Cognitive Decline and Dementia in Elderly Populations: A Meta-analysis of Prospective Cohort Studies. J. Gerontol. A Biol. Sci. Med. Sci..

[15] Verghese J., Wang C., Lipton R.B., Holtzer R. (2013). Motoric cognitive risk syndrome and the risk of dementia. J. Gerontol. A Biol. Sci. Med. Sci..

[16] Sheridan P.L., Solomont J., Kowall N., Hausdorff J.M. (2003). Influence of executive function on locomotor function: Divided attention increases gait variability in Alzheimer’s disease. J. Am. Geriatr. Soc..

[17] Demnitz N., Esser P., Dawes H., Valkanova V., Johansen-Berg H., Ebmeier K.P., Sexton C. (2016). A systematic review and meta-analysis of cross-sectional studies examining the relationship between mobility and cognition in healthy older adults. Gait. Posture..

[18] Kikkert L.H.J., Vuillerme N., van Campen J.P., Hortobagyi T., Lamoth C.J. (2016). Walking ability to predict future cognitive decline in old adults: A scoping review. Ageing Res. Rev..

[19] Montero-Odasso M., Almeida Q.J., Bherer L., Burhan A.M., Camicioli R., Doyon J., Fraser S., Muir-Hunter S., Li K.Z.H., Liu-Ambrose T. (2019). Consensus on Shared Measures of Mobility and Cognition: From the Canadian Consortium on Neurodegeneration in Aging (CCNA). J. Gerontol. A Biol. Sci. Med. Sci..

[20] Alexander N.B., Ashton-Miller J.A., Giordani B., Guire K., Schultz A.B. (2005). Age differences in timed accurate stepping with increasing cognitive and visual demand: A walking trail making test. J. Gerontol. A Biol. Sci. Med. Sci..

[21] Yamada M., Ichihashi N. (2010). Predicting the probability of falls in community-dwelling elderly individuals using the trail-walking test. Environ. Health Prev. Med..

[22] Klotzbier T.J., Schott N. (2017). Cognitive-Motor Interference during Walking in Older Adults with Probable Mild Cognitive Impairment. Front. Aging Neurosci..

[23] Perrochon A., Kemoun G. (2014). The Walking Trail-Making Test is an early detection tool for mild cognitive impairment. Clin. Interv. Aging.

[24] Osuka Y., Kojima N., Sakurai R., Watanabe Y., Kim H. (2020). Reliability and construct validity of a novel motor-cognitive dual-task test: A Stepping Trail Making Test. Geriatr. Gerontol. Int..

[25] Sugishita M., Koshizuka Y., Sudou S., Sugishita K., Hemmi I., Karasawa H., Ihara M., Asada T., Mihara B. (2018). The validity and reliability of the Japanese version of the Mini-Mental State Examination (MMSE-J) with the original procedure of the Attention and Calculation Task (2001). Jpn. J. Cogn. Neurosci..

[26] Folstein M.F., Folstein S.E., McHugh P.R. (1975). “Mini-mental state”. A practical method for grading the cognitive state of patients for the clinician. J. Psychiatr. Res..

[27] Tsoi K.K., Chan J.Y., Hirai H.W., Wong S.Y., Kwok T.C. (2015). Cognitive Tests to Detect Dementia: A Systematic Review and Meta-analysis. JAMA Intern. Med..

[28] Shinkai S., Watanabe S., Kumagai S., Fujiwara Y., Amano H., Yoshida H., Ishizaki T., Yukawa H., Suzuki T., Shibata H. (2000). Walking speed as a good predictor for the onset of functional dependence in a Japanese rural community population. Age Ageing.

[29] (1944). Army Individual Test Battery.

[30] Sanchez-Cubillo I., Perianez J.A., Adrover-Roig D., Rodriguez-Sanchez J.M., Rios-Lago M., Tirapu J., Barcelo F. (2009). Construct validity of the Trail Making Test: Role of task-switching, working memory, inhibition/interference control, and visuomotor abilities. J. Int. Neuropsychol. Soc..

[31] Yatomi N. (1994). The factor structure and item characteristic of the GDS (Geratric Depression Scale) short version in a Japanese elderly sample. Jpn. J. Gerontol..

[32] Swets J.A. (1988). Measuring the accuracy of diagnostic systems. Science.

[33] Pencina M.J., D’Agostino R.B., D’Agostino R.B., Vasan R.S. (2008). Evaluating the added predictive ability of a new marker: From area under the ROC curve to reclassification and beyond. Stat. Med..

[34] Steyerberg E.W., Vickers A.J., Cook N.R., Gerds T., Gonen M., Obuchowski N., Pencina M.J., Kattan M.W. (2010). Assessing the performance of prediction models: A framework for traditional and novel measures. Epidemiology.

[35] Allan L.M., Ballard C.G., Burn D.J., Kenny R.A. (2005). Prevalence and severity of gait disorders in Alzheimer’s and non-Alzheimer’s dementias. J. Am. Geriatr. Soc..

[36] Montero-Odasso M., Bergman H., Phillips N.A., Wong C.H., Sourial N., Chertkow H. (2009). Dual-tasking and gait in people with mild cognitive impairment. The effect of working memory. BMC Geriatr..

[37] Montero-Odasso M., Verghese J., Beauchet O., Hausdorff J.M. (2012). Gait and cognition: A complementary approach to understanding brain function and the risk of falling. J. Am. Geriatr. Soc..

[38] Montero-Odasso M.M., Sarquis-Adamson Y., Speechley M., Borrie M.J., Hachinski V.C., Wells J., Riccio P.M., Schapira M., Sejdic E., Camicioli R.M. (2017). Association of Dual-Task Gait With Incident Dementia in Mild Cognitive Impairment: Results From the Gait and Brain Study. JAMA Neurol..

[39] Rosso A.L., Studenski S.A., Chen W.G., Aizenstein H.J., Alexander N.B., Bennett D.A., Black S.E., Camicioli R., Carlson M.C., Ferrucci L. (2013). Aging, the central nervous system, and mobility. J. Gerontol. A Biol. Sci. Med. Sci..

